# On Multi-Parameter Optimization and Proactive Reliability in 5G and Beyond Cellular Networks

**DOI:** 10.3390/s25247651

**Published:** 2025-12-17

**Authors:** Aneeqa Ijaz, Waseem Raza, Sajid Riaz, Ali Imran

**Affiliations:** 1 AI4Networks Research Center, Department of Electrical & Computer Engineering, University of Oklahoma, Norman, OK 73019, USA; waseem@ou.edu (W.R.);; 2 James Watt School of Engineering, University of Glasgow, Glasgow G12 8QQ, UK

**Keywords:** fault prediction, conflict avoidance, Discrete Time Markov Chain (DTMC), reliability, misconfiguration, outage detection

## Abstract

Ultra-dense heterogeneous cellular networks in 6G and beyond face an escalating vulnerability to cell outages stemming from complex issues like parameter misconfigurations, hidden conflicts among Autonomous Network Functions (ANFs), multivendor incompatibility, and software/hardware failures. While ANF-based automated fault detection is a core capability for next-generation networks, existing solutions are predominantly reactive, identifying faults only after reliability is compromised. To overcome this critical limitation and maintain high service quality, a proactive fault prediction capability is essential. We introduce a novel Discrete-Time Markov Chain (DTMC)-based stochastic framework designed to model network reliability dynamics. This framework forecasts the transition of a cell from normal operation to suboptimal or degraded states, offering a crucial shift from reactive to proactive fault management. Our model rigorously quantifies the effects of fault arrivals, estimates the fraction of time the network remains degraded, and, uniquely, identifies sensitive parameters whose misconfigurations pose the most significant threat to performance. Numerical evaluations demonstrate the model’s high applicability in accurately predicting both the timing and probable causes of faults. By enabling true anticipation and mitigation, this framework is a key enabler for significantly reducing the cell outage time and enhancing the reliability and resilience of next-generation wireless networks.

## 1. Introduction

The forthcoming 6G and beyond (6G&B) wireless networks are expected to deliver massive enhancements in capacity, connectivity, and resilience to support new verticals from smart cities and Industry 4.0 to future autonomous systems [1,2]. In unlocking the hallmark capabilities of such wireless communication, manifestation of immensely resilient wireless networks is needed [3]. The 6G&B networks are envisaged to be a multi-service network supporting a wide range of verticals, for instance, network slicing [4], mmWave [5], network virtualization [6], to name a few, with a diverse set of performance and service requirements [7]. However, with the provision of heterogeneous services and gigabit wireless connectivity, the cellular networks are encountering numerous challenges related to the enhanced complexity, reliability [8], scalability, traffic management, latency, spectrum availability [9], load balancing [10], security [11], etc.

**Figure 1:**
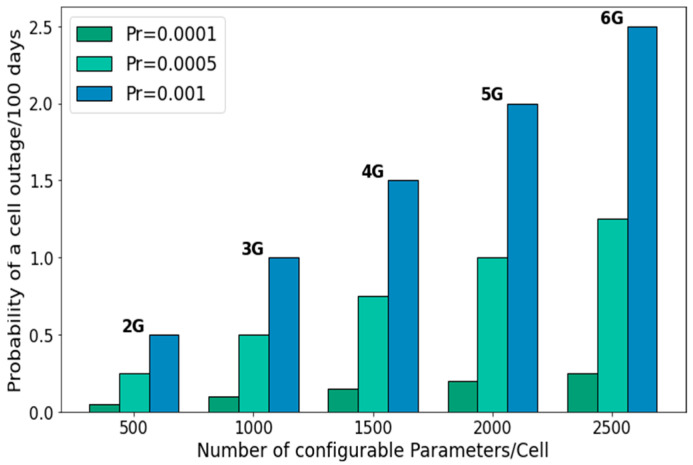
Probability of single parameter misconfiguration with the increase in configurable parameters.

Among the aforementioned issues, a key challenge is the ever-increasing complexity and densification of wireless networks. As we progress from 2G to 6G, the number of configurable parameters per site has grown steadily from around 500 in 2G to nearly 2500+ in 6G, while the density of sites per unit area continues to rise at a similar pace. As illustrated in Figure 1, this growth in configuration parameters significantly amplifies the probability of cell outages, thus can lead to a disproportionately higher chance of misconfiguration and subsequent service disruption. The parameter misconfiguration probability is derived using the Poisson-distribution-based method of the failure estimation [12]. Thus, 6G promises to meet the enhanced mobile broadband (eMBB) needs but at the cost of increased complexity, ultra dense networks and heterogeneous service requirements [13,14]. These factors can elevate the risk of network performance degradation, in terms of system level reliability [15]. Concurrently, the current manual operation of complex dense networks is prone to human-error-based parameter misconfiguration or suboptimal performance that becomes a bottleneck in achieving the network reliability [16]. Consequently, even in 5G, the network reliability is less than 98.78% [17]. In contrast, most emerging networks key use cases such as vehicle-to-everything (V2X) networks, smart grids, mission-critical applications such as real-time sensing, diagnostics and tactile robots, autonomous cars, UAVs, and remote surgery, to name a few, require reliability of greater than 99.9999% [18].

To overcome these challenges, the paradigm of Self-Organizing Network (SON)/Autonomous Network Functions (ANF) has emerged to automate the network configuration, management, and prognostic tasks [19]. SON functionalities were standardized by 3GPP release 10 and continue to be enhanced for 5G and beyond [20]. The automated functions of the SON/ANF networks are commonly classified as (1) self-configuring, (2) self-optimizing, and (3) self-healing functions [21]. The standardized SON functions include Inter-Cell Interference Coordination (ICIC), Coverage and Capacity Optimization (CCO), Mobility Load Balancing (MLB), Mobility Robustness Optimization (MRO), etc. For a detailed review of the ANF functions reader is referred to [22]. These network automation functions have self-adaption capabilities, in addition to the operational cost reduction and resource efficiency improvement [22]. However, despite all these auspicious ANF characteristics, there are challenges such as reliability, parametric and objective dependencies, data measurement and processing [21], unreliable ANF functions for mmWave-based cellular networks [23]. Moreover, as identified in [24,25,26], when operating concurrently, many SON/ANF functions are prone to a variety of hidden, logical, resource, policy and parametric conflicts. In addition to manual parameter misconfiguration, mis-specified intents in case of future intent-driven automated ANF networks [27], software/hardware failure, these faults can also lead to suboptimal network performance and eventually can cause partial or complete outages. Hence, spurring the seamless migration towards 5G&B networks demands the incorporation of new features and proactive algorithms for conflict coordination in the legacy ANF functions to render ultra reliability.

### 1.1. Related Work

A conflict/fault free ANF/SON orchestration is inevitable to enable reliable network operations. Thus, conflict avoidance in ANFs is a crucial yet understudied research problem. An earlier study [26] has identified a taxonomy to classify different conflicts such as KPI conflicts, network topology mutation conflicts, parameter conflicts, logical dependency conflicts, and measurement-based conflicts. The authors also presented several self-coordination mechanisms for the conflict resolution. In literature, the most extensively studied framework to avoid conflicts is the co-design conflict coordination algorithm [25,28,29,30,31]. Several studies have developed ANF conflict coordination schemes, in particular for the MRO and MLB SON functions. The authors in [26] suggested Trigger–Condition–Action (TCA) policies for the conflict coordination of the MLB and MRO functions. Another SON COordinator (SONCO) framework for the conflict resolution of the two distributing functions (MLB and MRO) is proposed in [32]. The reinforcement learning tunes the arbitrations, i.e., Cell Individual Offset (CIO) parameter for the MLB and handover hysteresis parameter for the MRO function on the basis of the operator assigned priorities. For the assurance of conflict-free operation of CCO and load balancing functions, an optimization problem is formulated to optimize the antenna tilt, transmit power, and CIO as a soft parameter in [29]. An operator’s objective-driven ANF coordination scheme is devised in [33] for three SON functions, namely, CCO, MLB, and MRO. The authors devised two coordination schemes, i.e., policy-based and objective-driven coordination. In the policy-based conflict resolution, CCO is being prioritized over MRO and MLB SON functions. Whereas, the objective-driven coordination determines the conflict-free subset of the requests that can prominently improve the utility and can select the concurrent execution of the two ANF functions. Thus, based on the multiattribute utility theory-based objectives, it is possible to achieve a conflict-free subset of the ANF function requests by solving a constraint optimization problem. Most of the proposed approaches in the literature consider conflict coordination specific to only two SON functions [25,26,28,29,31,32]. In [34] the authors proposed the enhancement of the Next Gen SON functions to improve the coverage and reliability of the 5G ultra dense network deployment in millimeter wave bands. The main idea is to configure the user to report the additional reflection environment map, that renders a fast recovery mechanism from the loss of connectivity caused by the poor channel conditions.

Despite the reviewed advancements, the state-of-the-art modus operandi is reactive, i.e., the fault detection and compensation mechanisms for automation conflicts are activated once the fault/outage has already occurred. Hence, even if misconfiguration/fault is detected, a disruption in service in the order of several seconds to minutes is inevitable. Therefore, without transforming the way conflicts are managed in the emerging networks, achieving high reliability may remain an elusive goal in 5GB networks [35]. In this paper, we propose a generalized fault prediction framework that can enable this transformation in the way automation caused or otherwise faults are handled in the network. The proposed model, unlike the state-of-the-art, is not limited to the structural aspects of the cells but takes into account the impact of parameter misconfiguration for the network fault scenarios. Our proposed framework not only characterizes the reliability of the automation enabled networks, but it can also predict future network behavior in terms of normal or suboptimal (partial/complete outage) performance to improve the network reliability. A network equipped with state-of-the-art automated but still reactive fault detection solution can offer more reliability than a network with manual operation by reducing the potential outages time from hours to minutes. However, with state-of-the-art reactive fault detection or post-conflict reliability enforcement, network cannot offer Ultra Reliable Low Latency Communications (URLLC), a major and yet to be realized 5G and beyond use case. In contrast, the proposed framework, by predicting the faults or automation conflicts before they happen, can enable preemptive maintenance, thereby significantly reducing the cell outage time and enabling proactive fault avoidance. Thus, the proposed solution offers a novel proactive approach that can act as a key enabler to the URLLC when it comes to fault detection or ensuring network reliability.

A detailed comparison of our proposed solution with the relevant solutions in the state-of-the-art literature is given in Table 1. More specifically, the presented model can predict the expected time and cause (parameter misconfiguration) of the suboptimal behavior by considering the expected conflicts among primary parameters. No existing solution offers such capability as explained above. Such capabilities can be leveraged for detection and isolation of early developing faults in the cellular networks as further explained in Section 4.

For the prediction of the future trends in wireless networks such as traffic and fault prediction, Machine Learning (ML) is being leveraged in recent years [23,28,30,31,36,37]. Fault prediction using ML has two possible approaches: (1) using historic network fault logs to create a regression or Deep Learning (DL) model [28,30,37], and (2) creating a state space Markov chain model and predicting the next states. For the purpose of prediction of future faults, the first approach requires large amounts of historical data. In contrast, the second approach can work with small amounts of historical data, as the data are only needed for initializing the transition probabilities. However, it does require more domain-knowledge-aware mathematical modeling of network parameters and behavior. Benefiting from the domain knowledge, our proposed proactive fault prediction framework leverages the latter approach. The additional benefits of the proposed solution include needing far less historical data to create the model, low computational complexity, and ability to incorporate domain knowledge that in turn can capture live network behavior that deep-learning-based black box models for fault prediction may miss.

The authors in [23] explored a proactive mobility and blockage management mechanism for the mmWave small cell ANF networks. They developed a big-data-empowered proactive ANF framework that leverages ML for the cell outage detection schemes. In [28] an ML-based zero-touch coordination framework is proposed to automatically learn the dynamics based on the history between the selected ANF functions (MRO and MLB) for the network optimization. In [30], ML is used for the network performance prediction of the SON functions (MLB, MRO) based on the network history. Subsequently, these predictions are used as inputs to a multi-objective optimization process, which searches for a set of non-dominated solutions or Pareto front. The authors concluded that the proposed solutions cannot improve the performance of one ANF function without degrading the other one. An ML-based zero-touch coordination framework to automatically learn the dynamics based on history between the selected SON functions (MRO and MLB) that assists the network optimization task is proposed in [28].

### 1.2. Contributions

In this paper, we propose a fault/misconfiguration prediction framework by using geometrical distribution for DTMC that can detect the upcoming network performance degradation. As mentioned earlier, there can be several reasons for network faults/outages. However, for the tractability of modeling the multi-parameter correlation—the central novelty of this work—we assume that the possible faults are predominantly due to the misconfiguration of network automation function parameters. This focus allows us to specifically and tractably model the stochastic correlation between multiple overlapping configuration parameters, which is a critical yet unaddressed source of network unreliability. Thus, the framework can allow the operator to enable them for the proactive maintenance of the suboptimal networks. The key insights gained from the presented analysis can be exploited to either design the predictive self-healing functions or preemptively schedule the parameter configuration and status checking routines to avoid the degraded performance altogether. The proposed outage/fault prediction model for conflict avoidance thus takes a step towards the reliable 5G&B networks. The contributions of this paper can be summarized as follows:

(1) By leveraging a realistic and generalized model, we formulate the probability of the network parameters to become suboptimal as a consequence of manual operations or network automation function conflicts. The misconfiguration probabilities for the given parameters are evaluated by the proposed reliability analysis to optimize the troubleshooting process.
(2) We present the stochastic analysis for the evaluation of long-term network behavior by exploiting Discrete Time Markov Chain (DTMC). The analysis is performed using four of the most impactful network parameters in terms of coverage reliability. We demonstrate through the state transition models that the small deviation in the values of these parameters can have a significant impact on the user quality of experience (QoE). Also, it is worth mentioning that our proposed model is general and can be applicable to other parameters.
(3) We formulate a tradeoff between the design parameters which overlap in multiple network automation and demonstrate that misconfiguration of primary parameters can cause the network performance degradation with the highest probability. For the proactive maintenance procedures, this model can be used to prioritize and schedule the parameter configuration verification.
(4) To observe the impact of the multi-parametric conflicts in network automation function, we devise two performance metrics, i.e., *mean first passage time and limiting or steady-state distribution*. By leveraging these metrics, we can evaluate the probability of the network degradation and perform the multi-parameter optimization.

The rest of the paper is organized as follows: Section 2 describes the reliability probability analysis of the ANF conflicts and the DTMC model. The performance evaluation and results are presented in Section 3. Section 4 describes the utility of the proposed fault prediction model. Finally, Section 5 concludes the paper.

## 2. Analysis

**Figure 2:**
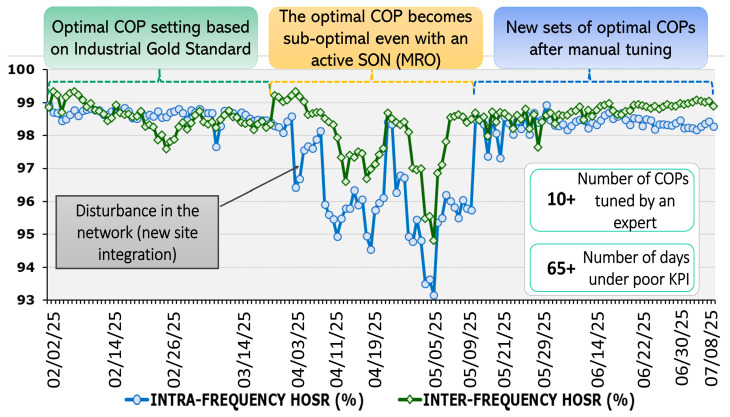
Suboptimal configuration of the Network Automation Function parameter.

In the network automation, there are several hundreds of configuration parameters that are related to ANF/SON, yet the majority of them are rarely changed. However, among these parameters, some are considered as the crucial parameters that belong to the multiple network automation functions and have a higher probability of being reconfigured. Some of the important ANF and their related configuration parameters are shown in Table 2. In general, the categorization of a parameter as a primary or secondary parameter can be performed based on the network logs, expert domain knowledge or by leveraging ML-based SHAP analysis. As mentioned earlier, the hidden, logical, parametric or objective-based conflicts between the network automation function parameters can increase the risk of parameter misconfiguration and can raise concerns regarding the network reliability. Therefore, we analyze the probabilistic reliability behavior of the 5G&B networks considering the conflicting ANF functions and their associated parameters. The misconfiguration of the network automation function parameters can degrade the performance of the entire network, i.e., when the network undergoes suboptimal settings, it requires time to detect the underlying cause, to activate the compensation actions, and consequently to stabilize the network. Figure 2 demonstrates a real network-based example of the misconfiguration of the MRO function. The data were collected from a mobile network operator (MNO) during the period of February–July 2025. It can be observed from the figure that the number of successful handovers within the same frequency (intra HO) and with different operating frequency (inter HO) is quite high when the Configuration and Optimization Parameters (COPs) are optimal based on the industrial Gold Standards. However, during the start of April, there is a prominent performance degradation in the MRO SON function due to the suboptimal value of one of its parameters, i.e., very high cell individual offset (CIO) value towards the neighboring cell of the serving node, that caused a hindrance in performing the handovers. The problem was eventually detected and solved by the careful configuration of more than 10 COPs with the help of human intervention. This example illustrates that the misconfiguration of a parameter can have a deteriorating impact on network reliability. The detection of such anomalous behavior requires highly trained domain experts to manually parse through the data logs. Hence, for the timely reliability provision, there is a need to analyze the probability of misconfiguration to enable proactive parametric conflict avoidance solutions in 5G cellular networks.

### 2.1. Assumptions

For the modeling of a proactive conflict avoidance network model, we focus the analysis specifically on the reliability impact of parameter misconfiguration, a primary source of automation-induced faults and take the following assumptions into account:

- A cell or node is defined as being in a suboptimal state (i.e., performance degradation or partial outage) if at least one of its network configuration parameters is set to a suboptimal value. This premise is based on the fact that the misconfiguration of even a single primary or secondary parameter can significantly degrade the network’s key performance indicators (KPIs), as illustrated by the real-network example in Figure 2.
- When an ANF is called (reactively or proactively), it will reconfigure only one of its associated parameters per call. This constraint is commonly adopted as an operational best practice for fault isolation and easier conflict resolution, preventing conflicts among interdependent parameters, which is a core focus of this work [33].

### 2.2. Model Development

**Figure 3:**
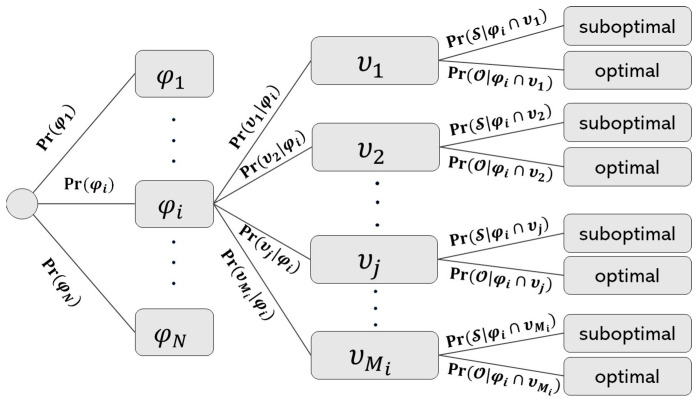
Probability Tree Diagram for ANF conflicts.

For the calculation of the misconfiguration probabilities, let us suppose we have *N* network automation functions *ϕ_N_* = {*ϕ*_1_, …, *ϕ_N_*} and *M_i_* is the number of primary and/or secondary parameters *υ_M_i__* = {*υ*_1_, …, *υ_M_i__* }, configurable by a ANF. At any time instant, one ANF function is called and its probability is *Pr*(*ϕ_i_*). The probability that *υ_j_* parameter is reconfigured when the ANF *ϕ_i_* gets activated is given by the conditional probability *Pr*(*υ_j_*|*ϕ_i_*). Let *Pr*(S) be the probability of any of the network parameters to become misconfigured/suboptimal when a network automation function is called and *Pr*(O) = 1 − *Pr*(S) be the probability of *υ_j_* becoming optimal. Thereby, the conditional probability of the *υ_j_* parameter to become suboptimal given that the network automation function *ϕ_i_* is called and *υ_j_* parameter gets selected is given by; *Pr*(S|(*ϕ_i_* ∩ *υ_j_*). These dependent probabilities for the ANF conflicts can be represented by the probability tree diagram as shown in Figure 3.

Thus, the total probability of a network parameter to become suboptimal when the ANF is called can be given by [38]:

(1) $$\mathrm{Pr}\left(S\right)=\;\sum_{i=1}^{N}\sum_{j=1}^{M_{i}}\mathrm{Pr}\left(\phi_{i}\right)\times \mathrm{Pr}\left(\upsilon_{j}|\phi_{i}\right)\times \mathrm{Pr}\left(S|\phi_{i}\;\cap \upsilon_{j}\right)$$

Similarly, the probability of a single network parameter, for instance, the parameter handover *H* to become suboptimal when a ANF function gets activated is given as:

(2) $$\mathrm{Pr}\left(H_{S}\right)=\sum_{i=1}^{N}Pr\left(\varphi_{i}\right)\times Pr\left({H|\varphi }_{i}\right)\times Pr\left({S|\varphi }_{i}\cap H\right)$$

The probability of a function to get selected is:

(3) $$Pr(\varphi_{i})=\frac{1}{N},\;\;equally\;likely\;\forall i=1\;to\;N\;$$

Or they can have different probabilities such that $\sum_{i=1}^{N}Pr(\phi_{i})=1$.

The conditional probability $\mathrm{Pr}({\upsilon_{j}|\phi }_{i})$ of Equation (1) can be further classified as follows:

- If the network automation function *ϕ_i_* operates on all of its *M_i_* parameters with equal priority, then the probability of *υ_j_* given *ϕ_i_* gets called is: *Pr*(*υ_j_*|*ϕ_i_*) = ($\frac{1}{M}$).
- If the network automation function *ϕ_i_* has *M_pi_* and *M_si_* number of primary and secondary parameters, respectively, such that *M_i_* = *M_pi_* + *M_si_*, then the probability of *υ_j_* given *ϕ_i_* is:

(4) $$\mathrm{Pr}(\upsilon_{j}|\phi_{i})=\left\{\begin{array}{c} \left(\frac{\mathrm{Pr}\left(Primary\;parameter\right)}{M_{P_{i}}}\right)\;,\;\;\upsilon_{j}\;is\;primary\;prarameter\;of\;\phi_{i}\\ \left(\frac{\mathrm{Pr}\left(Secondary\;parameter\right)}{M_{s_{i}}}\right),\;\;\upsilon_{j}\;is\;secondary\;prarameter\;of\;\phi_{i} \end{array}\right.$$

Equation (1) is used when the network automation function is activated once. However, with the passage of time, as the network automation functions are activated repeatedly, we have the Bernoulli trials with two outcomes to model this scenario: suboptimal or optimal configuration of the parameters. Based on the Bernoulli trials assumptions, i.e., the trials are independent, each trial has one of the two possible outcomes, and the probability of the outcome remains constant from trial to trial [38]. We are considering that the ANF are called different times of the day and are independent. If the network automation function is activated *n* number of times, in that case, the probability of getting *r* successes is given as, nr pr(1−p)n−r .

As time progresses and the network automation functions are activated *r* number of times, the probability of having the optimal parameters in all activations is given by (1 − *Pr*(S))*^r^.* Therefore, the probability of getting at least one suboptimal parameter in *r* number of runs will be:

*Pr*(at least one subopt parm) = 1 − (1 − *Pr*(S))*^r^* (5)

where *Pr*(S) is calculated from Equation (1). The probabilities can be extracted from the real networks, e.g., Operations and Maintenance Centre (OMC) network logs that will have information on the frequency of the network automation functions activation.

### 2.3. Markov Analysis

DTMC can be used to model the long-term network behavior. It is a discrete time stochastic process on the state space *S*, where the conditional probability of any future event is only dependent upon the current state and is independent of the past states. Mathematically for all *y* and *z* in *S*:

(6) $$P\left(X_{t+1}=z\;\right|X_{t}=y,\;X_{t-1},\;\ldots ,\;X_{0})=P(X_{t+1}=z|\;X_{t}=y)\;$$

As in our network model, the transition conditional probabilities are not dependent upon time, therefore, the DTMC will be time homogeneous, and defined as:

(7) $$P\left(X_{t+1}=z\;\right|\;X_{t}=y)=P\left(X_{t}=z\;\right|\;X_{t}=y)=p_{yz}$$

where *p_yz_* is a one-step transition probability from state *y* at time *t* to state *z* at time *t* + 1. For a Markov Chain with *L* number of states, the transition matrix **T** can be expressed as an *L* × *L* matrix:

(8) $$T=\left[\begin{array}{ccccc} p_{11} & p_{12} & p_{13} & \ldots & p_{1L}\\ p_{21} & p_{22} & p_{23} & \ldots & p_{2L}\\ p_{31} & p_{32} & p_{33} & \ldots & p_{3L}\\ \ldots & \ldots & \ldots & \ldots & \ldots \\ p_{L1} & p_{L2} & p_{L2} & \ldots & P_{LL} \end{array}\right]$$

### 2.4. Transition Matrix Properties

For the purpose of our analysis, we have chosen four network parameters: “P—Downlink Tx Power, A—Antenna Tilt and Azimuth, H—Handover Parameters, and M—MIMO Configuration”, that intuitively have the maximum impact on the coverage reliability^3^. This set represents a high-impact, conflict-prone subset chosen to demonstrate the framework’s capability to model joint configuration dynamics, although the model is algebraically applicable to any number of parameters (*L*) affecting different QoS dimensions. A small deviation in their values can have a significant impact on the user experience [35]. The discrete time t corresponds to the activation of the ANF for the *t*th time. At any time instant, when a network automation function is called, any of these parameters can be in one of the two states, i.e., optimal or suboptimal. The following properties govern the transition matrix T and are used to set the necessary conditions aligned with the realistic assumptions:

- As the number of parameters is finite, i.e., four in our case, therefore, the DTMC State Space *S* will be finite, consisting of 24 = 16 states.

(9) $$S=\left[S_{1}{\;S}_{2}\;S_{3}\ldots S_{l}\right],\;\;l=16$$

- $p_{yz}$ is a positive real number between 0 and 1

(10) $${0\leq p}_{yz}\leq \;1\;\forall \;y,\;z\;\in [1,\;L]$$

- T will be a right stochastic matrix, i.e.,

(11) $$\sum_{z=1}^{L}p_{yz}=1,\;1\leq y\leq L.\;$$

### 2.5. Transition Matrix Initialization

It is assumed that whenever any network automation function is activated, it operates on only one of its associated parameters per transition, this assumption is also supported by [39]. The transition probabilities are calculated as per Table 3. Therefore, those states cannot be reached directly (zero transition probability) in which more than one parameter is changed.

There will be a total of 16 DTMC States as defined in Table 4, wherein ✓ indicates that the parameter is configured with the optimal value while ✗ represents the misconfigured value. Using the information in Table 2 and Table 3, the Transition matrix **T** is given as:

(12) $$T=\left[\begin{array}{cccccccccccccccc} \Psi & m^{\prime } & h^{\prime } & 0 & a^{\prime } & 0 & 0 & 0 & p^{\prime } & 0 & 0 & 0 & 0 & 0 & 0 & 0\\ m & \Psi & 0 & h^{\prime } & 0 & a^{\prime } & 0 & 0 & 0 & p^{\prime } & 0 & 0 & 0 & 0 & 0 & 0\\ h & 0 & \Psi & m^{\prime } & 0 & 0 & a^{\prime } & 0 & 0 & 0 & p^{\prime } & 0 & 0 & 0 & 0 & 0\\ 0 & h & m & \Psi & 0 & 0 & 0 & a^{\prime } & 0 & 0 & 0 & p^{\prime } & 0 & 0 & 0 & 0\\ a & 0 & 0 & 0 & \Psi & m^{\prime } & h^{\prime } & 0 & 0 & 0 & 0 & 0 & p^{\prime } & 0 & 0 & 0\\ 0 & a & 0 & 0 & m & \Psi & 0 & h^{\prime } & 0 & 0 & 0 & 0 & 0 & p^{\prime } & 0 & 0\\ 0 & 0 & a & 0 & h & 0 & \Psi & m^{\prime } & 0 & 0 & 0 & 0 & 0 & 0 & p^{\prime } & 0\\ 0 & 0 & 0 & a & 0 & h & m & \Psi & 0 & 0 & 0 & 0 & 0 & 0 & 0 & p^{\prime }\\ p & 0 & 0 & 0 & 0 & 0 & 0 & 0 & \Psi & m^{\prime } & h^{\prime } & 0 & a^{\prime } & 0 & 0 & 0\\ 0 & p & 0 & 0 & 0 & 0 & 0 & 0 & m & \Psi & 0 & h^{\prime } & 0 & a^{\prime } & 0 & 0\\ 0 & 0 & p & 0 & 0 & 0 & 0 & 0 & h & 0 & \Psi & m^{\prime } & 0 & 0 & a^{\prime } & 0\\ 0 & 0 & 0 & p & 0 & 0 & 0 & 0 & 0 & h & m & \Psi & 0 & 0 & 0 & a^{\prime }\\ 0 & 0 & 0 & 0 & p & 0 & 0 & 0 & a & 0 & 0 & 0 & \Psi & m^{\prime } & h^{\prime } & 0\\ 0 & 0 & 0 & 0 & 0 & p & 0 & 0 & 0 & a & 0 & 0 & m & \Psi & 0 & h^{\prime }\\ 0 & 0 & 0 & 0 & 0 & 0 & p & 0 & 0 & 0 & a & 0 & h & 0 & \Psi & m^{\prime }\\ 0 & 0 & 0 & 0 & 0 & 0 & 0 & p & 0 & 0 & 0 & a & 0 & h & m & \Psi \end{array}\right]$$

wherein $\Psi =1-\;\sum_{y\neq z}a_{yz},\;\;\forall \;z,\;\;a_{yz}\in T$ to satisfy right stochastic property.

Where Ψ represents the probability of the network remaining in its current state $S_{y}$ (i.e., the diagonal element $p_{yy}$) and is mathematically defined to satisfy the right stochastic matrix property (Equation (11)) such that:

$$\Psi =p_{yy}=1-\sum_{z\neq y}p_{yz}$$

**Table 4:** Discrete time Markov chain (DTMC) states.

States	Parameters Configuration ✓: Optimal ✗: Suboptimal			
	P	A	H	M
*S*_1_	✓	✓	✓	✓
*S*_2_	✓	✓	✓	✗
*S*_3_	✓	✓	✗	✓
*S*_4_	✓	✓	✗	✗
*S*_5_	✓	✗	✓	✓
*S*_6_	✓	✗	✓	✗
*S*_7_	✓	✗	✗	✓
*S*_8_	✓	✗	✗	✗
*S*_9_	✗	✓	✓	✓
*S*_10_	✗	✓	✓	✗
*S*_11_	✗	✓	✗	✓
*S*_12_	✗	✓	✗	✗
*S*_13_	✗	✗	✓	✓
*S*_14_	✗	✗	✓	✗
*S*_15_	✗	✗	✗	✓
*S*_16_	✗	✗	✗	✗

The off-diagonal non-zero transition probabilities (p′, a′, h′, m′, p, a, h, m) used in T are explicitly calculated using the generalized total probability formula (Equation (1)), as detailed in Table 3.

### 2.6. Transition Matrix Analysis

Using the Chapman–Kolmogorov equation, the probability of the network moving from state *y* to state *z* after *k* periods is calculated by multiplying the transition matrix itself k times. If *p*_0_ is the initial probability state vector, then

(13) $$p_{k}=p_{0}T^{k}$$

where $p_{0}=[p_{1}{\;p}_{2}\;p_{3}\ldots p_{L}]$ is the initial distribution vector and $p_{k}=[\gamma_{1}{\;\gamma }_{2}\;\gamma_{3}\ldots \gamma_{L}]$ is the *k*th probability vector for *l* states.

In order to find out the number of transitions after which the network first becomes suboptimal for each state of the DTMC, we define mean first passage time performance metric, i.e., expected time for the first occurrence of fault. The mean first passage time provides a measure of the network to be suboptimal due to each of the parameter’s misconfiguration status connected to the network automation functions [40]. The mean first passage time from state *y* to state *z* is the expected number of transitions before the network first reaches to the state *z*, given that the network is currently in state *y*. Denoted by *m_yz_*, it is evaluated as:

(14) $$m_{yz}=1+\;\sum_{k\neq j}p_{yk}m_{zj}\;$$

The limiting or steady-state distribution is a measure to indicate the fraction of time the network will be in each of the states [37]. It is defined as:

π = [π_1_, π_2_, π_3_, …, π_l_] (15)

where

(16) $$\pi_{z}=\underset{t\to \infty }{\lim}P\left(X_{t}=z\right),\;z\;\in S$$

This steady-state distribution can also be used to find the cost of having suboptimal behavior. The long run expected loss due to unreliable network behavior can be calculated as:

(17) $$Revenue\;Loss=\;\sum \pi_{z}c\left(z\right)\;\forall \;z\;\in Sub-optimal\;States$$

where *c*(*z*) is the operator-defined loss (cost) incurred due to suboptimal network behavior in state z and $\pi_{z}$ is the final state distribution. This approach allows operators to leverage the DTMC analysis to perform a customized business-case assessment based on their specific service level agreements (SLAs) and penalty structures.

## 3. Numerical Results

For the numerical analysis, we consider the network settings of the network automation functions and their associated parameters as described in Table 2. The initial assumed probabilities for the numerical analysis are given in Table 5, unless stated otherwise. These assumptions are required because, while the authors have extensive experience working with real-world network traces, the proprietary nature of operator logs prohibits the publication of the exact statistical distributions of ANF invocation and misconfiguration rates. Therefore, the results presented in this section serve as a numerical illustration of the model’s analytical power and prescriptive utility, demonstrating its potential to derive metrics like first passage time and steady-state reliability, rather than providing an empirical validation of a specific real-world deployment.

**Figure 4:**
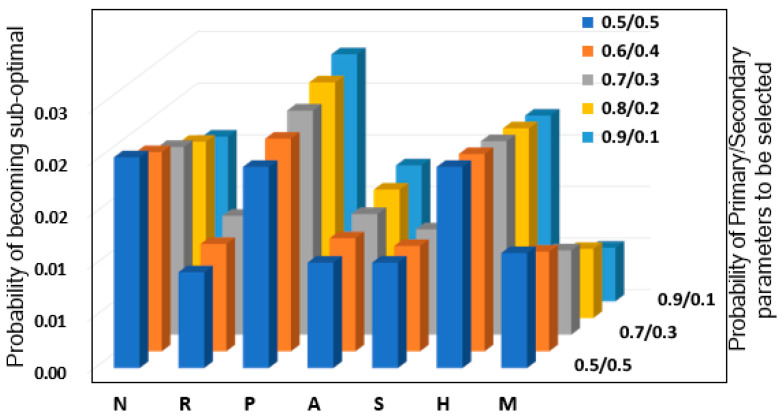
Multi-Parameter Conflict Risk Analysis: Probability of individual parameters becoming suboptimal as a function of selection priority (primary vs. secondary), demonstrating highest risk for overlapping parameters (P and A).

In Figure 4 we plot the probability of the network parameters to become suboptimal when configured for the different network automation functions. For this figure, we leverage Equation (1) with different probability of selection of the primary and secondary parameters. The result conforms to the intuitive notion that the overlapping parameters in multiple network automation functions as primary parameters have the highest probability to become suboptimal.

The power parameter *P* (a primary parameter for six network automation functions in Table 1) has the highest probability of misconfiguration followed by the handover *H* and neighbor list *N* parameters. In addition, it is worth noting that there is an increasing trend in the misconfiguration probability for some parameters as the probability of the primary parameter to be selected is increased from 0.5 to 0.9, while some parameters have decreasing trends. For instance, MIMO Configuration *M* parameter in Table 2 is listed mostly as a secondary parameter and with the decreasing probability of secondary parameters to be selected along z-axis, the probability of such parameters to become suboptimal is reduced. While the parameters which appear mostly as the primary parameters e.g., power *P*, antenna tilt and azimuth *A*, these parameters show increasing trends across the z-axis. Thus, the parametric misconfiguration probabilities that are calculated by leveraging the proposed reliability analysis can be beneficial to prioritize the verification of each parameter based on its estimated misconfiguration probability.

**Figure 5:**
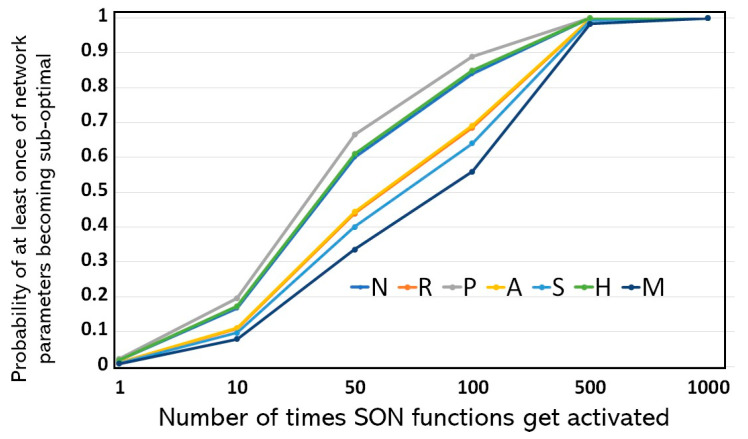
Predictive Scheduling Metric: Probability of experiencing at least one suboptimal parameter over the number of ANF activation cycles, providing a crucial time horizon for scheduling proactive maintenance.

The probability of the parameter misconfiguration against the number of times the network automation functions are activated is shown in Figure 5. We assume that all the ANF have the equal chances of being activated and the probability of each of the network parameters to become suboptimal is calculated using Equation (5). It is evident from Figure 5 that after 500 transitions, for the ANF activation, the network is expected to experience parameter misconfiguration with eminent high probability. Thus, the higher the number of times the network automation functions are activated, the chances of the parameters’ misconfiguration also escalates. This plot provides a measure for the proactive maintenance procedures to schedule the network parameter configuration verification after a certain number of transitions.

**Figure 6:**
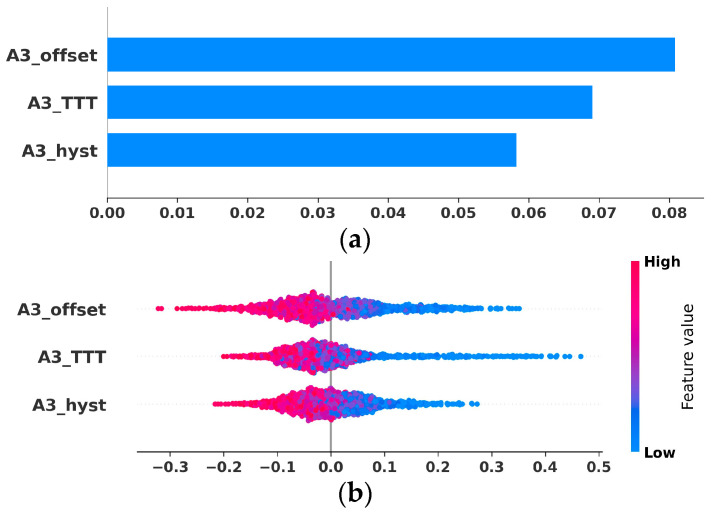
Parameter Sensitivity Analysis (SHAP): Empirical impact of MRO parameters (A3 offset, TTT, Hysteresis) on RSRP/network performance, justifying their classification as sensitive and high-priority inputs for the DTMC. (**a**) Impact of parameters on Mean RSRP value. (**b**) SHAP value (impact on model output).

In real wireless networks, some network automation functions are called more frequently compared to others, such as ICIC, MLB, and MRO network automation functions compared to ANR or PCI functions. Equation (1) and (2) state that the probability of selection of a particular network automation function has a direct impact on the misconfiguration of the parameters. Therefore, these ‘risky’ ANF which have a large number of ‘sensitive’ parameters should be scheduled relatively less frequently. Results in Figure 6 corroborate this contemplation. For the figure, we leverage a 3GPP-compliant system level 5G simulator [41]. A simulation area of 1.5 km x 1.5 km is used for data generation. User mobility type is random waypoint and the speed remains constant for a user. The details of the network level simulation parameters are summarized in Table 6. We emphasize that this simulation setup, though limited in scale (3 BSs and 50 Users), is used exclusively to generate empirical data for the SHAP analysis, which serves only to substantiate the selection and sensitivity ranking of the core DTMC parameters, rather than validating the DTMC framework’s performance metrics.

The figure illustrates that these three handover-related parameters have a varying impact on the network mean Reference Signals Received Power (RSRP) values. When we perform the Shapley analysis of the handover parameters, to analyze the sensitivity of uncertain variables, it can be observed that the A3 offset has the most substantial impact on the RSRP value and thus for triggering the handover as shown in Figure 6a,b. Therefore, it can be categorized as a sensitive parameter. This figure depicts the fact that for the SON functions such as MRO and MLB, where handover is the primary parameter, the configuration frequency of this parameter in overlapping ANF/SON functions should be less, to minimize the probability of misconfiguration. In reactive network automation functions, the probabilistic distribution of the activation of particular network automation depends upon the mobility and usage behavior of the users. However, in case of proactive SONs, frequency of different network automation functions depends upon the policies defined by the operator. As a result, network automation function scheduling can be viewed as a multi-objective optimization problem to determine the optimal scheduling such that the probability of the system becoming suboptimal is minimized.

To move beyond the initial generic assumptions (Table 6) and achieve empirical justification, the DTMC requires non-uniform inputs derived from network data. Specifically, the parameter selection probability ($Pr({\upsilon_{j}|\phi }_{i})$) can be justified by leveraging the sensitivity ranking (such as the SHAP values shown in Figure 6) or expert domain knowledge, assigning a higher selection probability to high-impact parameters. The misconfiguration probability $Pr\left(S\right|\phi_{i}\cap \upsilon_{j})$ is then empirically estimated as the historical ratio of observed configuration changes resulting in a suboptimal network state, calculated directly from network operation and maintenance center (OMC) logs. The analytical framework is designed to directly incorporate these non-uniform, empirically derived probabilities. The MRO network automation function tunes three mobility parameters related to the intra-frequency handover, i.e., A3 offset, A3 TTT, and A3 hysteresis to achieve the optimized objectives. A range of 2 dB to 15 dB is used for the A3 offset while for A3 TTT 7 values from 64 ms to 512 ms with an interval of 64 ms are used, and 1 dB to 10 dB range is considered for A3 hysteresis to generate the COP-KPI data. The figure illustrates that these three handover-related parameters have a varying impact on the network mean Reference Signals Received Power (RSRP) values. When we perform the Shapley analysis of the handover parameters, to analyze the sensitivity of uncertain variables, it can be observed that the A3 offset has the most substantial impact on the RSRP value and thus for triggering the handover as shown in Figure 6a,b. Therefore, it can be categorized as a sensitive parameter. This figure depicts the fact that for the SON functions such as MRO and MLB, where handover being the primary parameter, the configuration frequency of this parameter in overlapping ANF/SON functions should be less, to minimize the probability of misconfiguration. In reactive network automation functions, the probabilistic distribution of the activation of particular network automation depends upon the mobility and usage behavior of the users. However, in the case of proactive SONs, frequency of different network automation functions depends upon the policies defined by the operator. As a result, network automation function scheduling can be viewed as a multi-objective optimization problem to determine the optimal scheduling such that the probability of the system becoming suboptimal is minimized.

**Figure 7:**
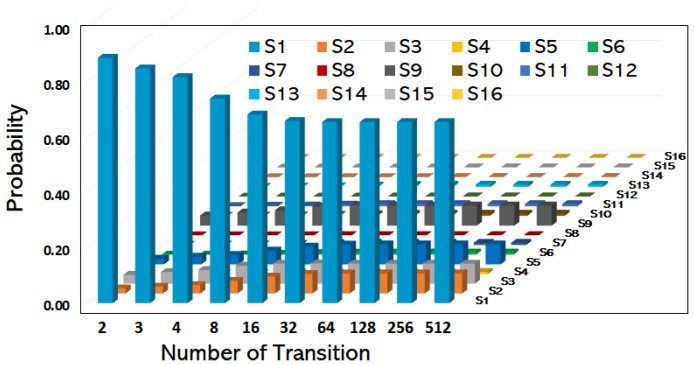
Transient Analysis and Convergence: Probability distribution across the 16 DTMC states over time, showing the rapid convergence to the steady-state distribution and providing the basis for Mean First Passage Time calculation.

Figure 7 shows the probability of each state against the number of times a network automation function is activated. With the assumption that the system is in state 1 at time *t* = 0 with the transition probabilities as prescribed in Table 3, the results for the state probabilities for 512 transitions of a network automation operation using the Chapman–Kolmogorov equation are shown. It is observed that after approximately 64 transitions, the network achieves the steady-state distribution, which is given as:

*π*_16_ = [0.6561, 0.0729, 0.0729, 0.0081, 0.0729, 0.0081, 0.0081, 0.0009, 0.0729, 0.0081, 0.0081, 0.0009, 0.0081, 0.0009, 0.0009, 0.0001].

The convergence to the steady state is determined when the $L_{1}$ norm (absolute difference) between successive probability vectors falls below a predefined tolerance threshold $\epsilon :\sum_{\;\left\{z=1\right\}}^{L}\left|p_{z}^{\left(k+1\right)}-p_{z}^{\left(k\right)}\right|\leq \epsilon$, where we use a threshold of $\epsilon =10^{-4}$ for this analysis. The fraction of times the network will be in each of the states is not changing after 64 transitions until 512 state transitions. As per the steady-state distribution, in the long run, the network remains in the optimal state 65.61% of the time and remains in the suboptimal state for 34.39% of the time.

**Figure 8:**
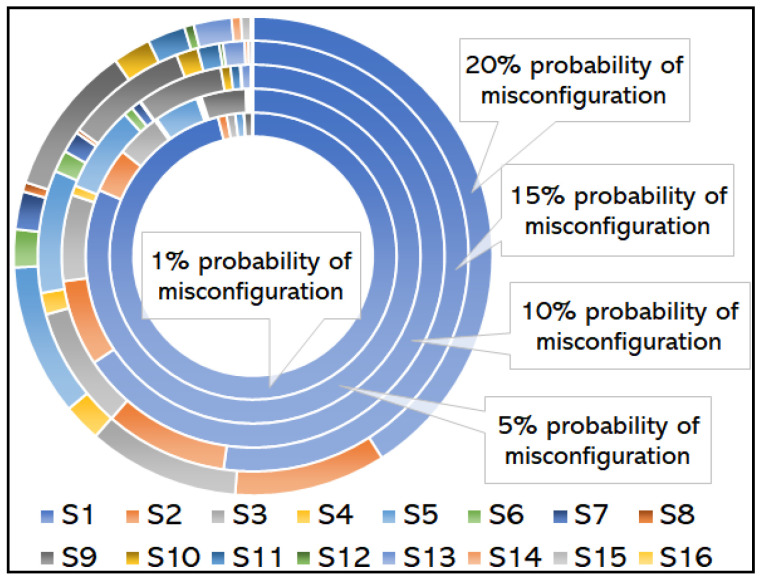
Long-Term Reliability Baseline: Steady-state distribution (π) illustrating the fraction of time the network remains in optimal (S1) versus suboptimal states as misconfiguration probability increases, establishing the long-term reliability ceiling.

We plot the steady-state probability distribution versus the probability of misconfiguration in Figure 8. The steady-state distributions, as defined earlier, depict the fraction of time for which the network will be in each of the states in the long term. Figure 8 shows different values of probability of suboptimality. The network will be in a stable state most of the time when there is 1% chance of parameter misconfiguration, i.e., the parameters are configured correctly with higher probability (96.06%) for different activated network automation functions in the network. However, as this misconfiguration probability is increased in steps from 1% to 5%, 10%, 15%, and 20%, i.e., most of the configurations result in transforming the network into suboptimal, the network optimal duration time reduces to 81.45%, 65.61%, 52.20% and 40.96%, respectively. This result demonstrates that the higher the parametric misconfiguration probability in a network automation function, the higher the chances are of the network to be in the outage/degraded performance. Additionally, when there is a 15% chance of parameter misconfiguration, approximately half of the time the network remains in the suboptimal state.

**Figure 9:**
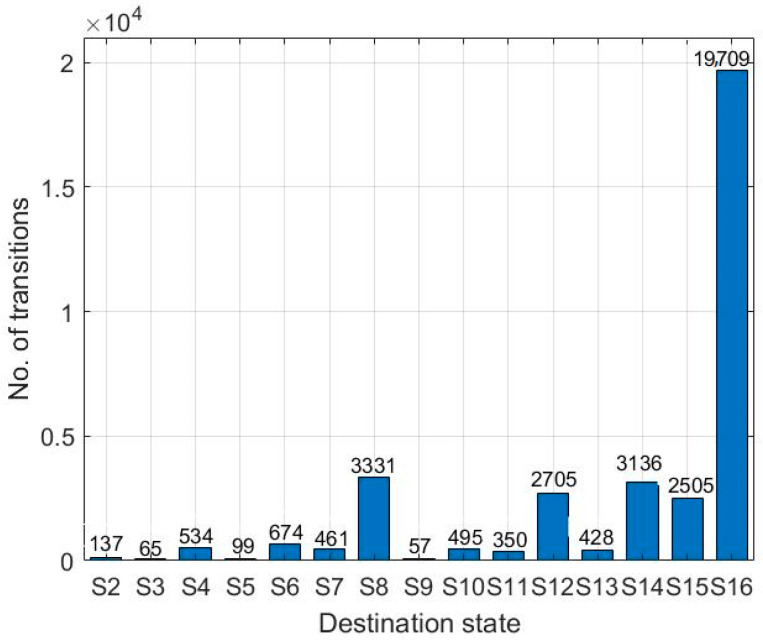
Proactive Fault Anticipation: Mean First Passage Time $m_{1z}$ from the optimal state ${(S}_{1})$ to all suboptimal states ${(S}_{2}-S_{16})$, providing the predicted transition count (lead time) before the first occurrence of a specific fault state.

Figure 9 shows the mean first passage time of DTMC from the optimal state *S*_1_ to all the other suboptimal states *S*_2_ − *S*_16_, which is calculated using Equation (14). The transitions state values vary from 57 (*S*_9_) to 19,709 (*S*_16_). As the minimum number of transitions from the optimal state *S*_1_ to the suboptimal state *S*_9_ is 57, it can be stated that for the first time after the launch, the network automation functions are optimized by configuring the parametric values correctly for the initial 56 transitions. However, the network is expected to undergo the suboptimal state immediately after 57 transitions. Thus, using this network performance metric, we can predict when the network is going to experience the suboptimal state for the first time.

## 4. Utility of the Proposed Framework

The continued increase from 2G to 5G in the complexity, densification, and the number of the configuration parameters per site lead to a higher risk of the network performance degradation. As to date, most of the network parameter optimization is performed manually, and the human error risk increases with the number of configurable parameters and the ultra dense networks. The current design of the network automation functions is prone to a variety of parametric, logical, and other type of conflicts that can cause the suboptimality of the network. Hence, the rate of the under-performance is proportional to the complexity and density of the network. Consequently, to achieve reliability, in addition to the physical layer innovations for the increased throughput, the emerging network needs resilient, proactive, and predictive fault/outage detection frameworks. The presented framework not only provides a tool for the reliability evaluation of the given network, but also can act as an enabler for the proactive preventive maintenance of the suboptimal networks. The model’s generalizability extends to non-radio parameters, such as transport slice bandwidth or core network user plane function (UPF) configuration settings. It can help to minimize the detection time of the fault/outage or avoid performance degradation altogether by preempting them.

**Figure 10:**
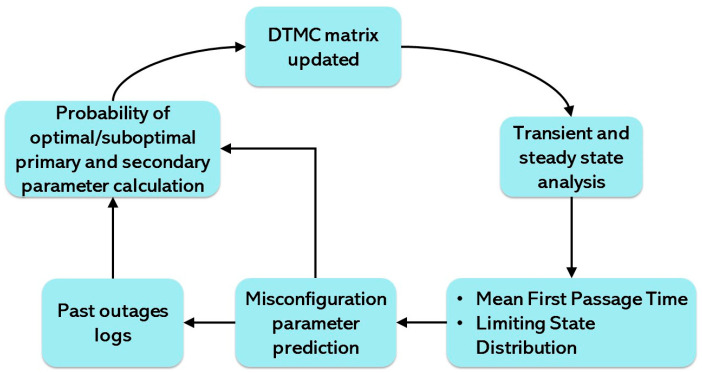
Parameter configuration scheduling prediction framework to avoid manual/network automation conflicts.

More specifically, the proposed framework predicts the expected time of the first occurrence of the fault and long-term reliability behavior of the network, as shown in Figure 10. By leveraging the proposed DTMC model and using the standard ML tools for initializing the transition probabilities, operators can leverage the Mean First Passage Time ($m_{yx}$) and Limiting State Distribution (π) to prioritize and schedule the proactive network maintenance verification and optimization as part of a multi-objective scheduling algorithm. The numerical results corroborate the fact that the primary parameters which overlap in the multiple network automation functions or manual optimization routines have the highest probability of misconfiguration. Hence, to enable predictive and prognostic managements that will reduce the risk of faults, frequent verification of these particular parameters should be scheduled. The results show that with the increasing probability of selection as a primary parameter, the probability of misconfiguration also increases. By leveraging the proposed reliability analysis framework, it is possible to calculate the first occurrence of misconfiguration probabilities for a given parameter to avoid faults/outages. Hence, we can prioritize the verification of each parameter based on its estimated misconfiguration probability.

The proposed model not only predicts the probabilities for the network to be in suboptimal condition, but operators can also leverage this model to evaluate the time the network may first enter (via Mean First Passage Time) and remain in (via Limiting State Distribution) the suboptimal state. Therefore, our proposed model provides a proactive fault detection framework that can be combined with existing solutions for outage compensation to enable complete proactive self-healing (fault/outage detection and compensation). This can be completed by preemptively activating the compensation mechanism as soon as any suboptimality is predicted in the network, utilizing the prediction lead time provided by the model’s transient analysis. Thus, network reliability higher than that enabled by current reactive outage detection and compensation solutions can be achieved by adopting such comprehensive proactive self-healing solutions in a 5G&B network.

## 5. Conclusions

We have introduced a Discrete-Time Markov Chain (DTMC)-based stochastic framework that fundamentally shifts fault management from reactive identification to proactive prediction in complex ANF-enabled networks. By modeling probabilistic reliability and integrating the unique impact of parameter misconfiguration, our model can anticipate the expected time and probable cause of outages well before service degradation occurs, allowing for preemptive measures that help in significantly reducing the cell outage time and enabling proactive fault avoidance. Numerical results validate the framework’s power in providing both a complete reliability analysis and pinpointing performance threats, establishing a crucial proactive mechanism for conflict-free SON orchestration. Future work will focus on refining the model’s parameter estimations using live network failure logs and expanding the DTMC state space to incorporate other major fault sources (e.g., hardware/software failures). This will evolve the framework into a more adaptive and resilient predictive system tailored for comprehensive real-world reliability analysis. We will also investigate using non-homogeneous DTMCs to capture the time-varying nature of ANF activations and non-stationary network conditions, thereby allowing the incorporation of parameters linked to broader QoS/URLLC KPIs by expanding the dimensionality (*L*) of the state space.

## Figures and Tables

**Table 1:** Comparison of state-of-the-art literature and our work.

Ref No.	ANF Function		Parameters	Contributions
Lateef et al. 2015 [25]	Combination of any 2 SON functions (CCO, ICIC, COC, MLB, etc.)		Antenna tilt, Tx power, CIO, TTT, hysteresis, filter co-efficient	Identification and classification of potential conflicts among the distinctive SON functions
Rojas et al. 2020 [28]	MLB, MRO		CIO, TTT, hysteresis	A machine learning framework for self adaption of SON function and network optimization once conflicts happened
Asghar et al. 2018 [29]	CCO, LB		Tx power, antenna tilt	Concurrent optimization of coverage, capacity, and load balancing SON functions in hetNETs through soft and hard cell association parameters.
Moysen et al. 2018 [30]	MLB, MRO		CIO, hysteresis, and TTT	A ML model and multi-objective algorithm that resolves SON conflicts and predicts network performance based on historical UE measurement
Bag et al. 2020 [31]	ICIC, CCO		Power factor, antenna tilt, edge to center boundary	Recommender System that determines coverage and capacity in different cells and recommends configuration parameters to maximize the multi-objective performance of both SON functions
Iacoboaiea et al. 2014 [32]	MRO, MLB		CIO, hysteresis	Reinforcement learning framework SONCO to resolve conflicts by jointly optimizing both SON functions based on the operator priorities
Frenzel et al. 2015 [33]	CCO, MLB, MRO		CQI, CIO	The conflict resolution of objective-driven SON functions by giving CCO SON function the highest priority compared to the MLB and MRO. The SON objective manager concept is modeled as a constraint optimization problem
Our work	Combination of ANF functions CCO, ICIC, EE, etc.)	any 4 (MLB, MRO,	Neighbor list, Radio bearer assignment, Downlink Tx power, antenna tilt and azimuth, switching on/off cell, handover parameters (CIO, hysteresis, and TTT), MIMO configuration	Discrete Time Markov Chain (DTMC analysis is performed using four of the most impactful network parameters in terms of coverage reliability, thus joint optimization of 4 ANF functions

**Table 2:** SON/ANF Functions and related parameters.

ANF Function	Description	Primary Parameters	Secondary Parameters
ANR/PCI	ANR automatically builds and maintains neighbor relationships. PCI automatically configures Physical Cell Identity of an eNB	*N*	-
ICIC	Reduces intercell interference	*R*	*P*, *A*, *S*
Coverage hole management	Automatically detects and compensates coverage holes in timely manner	*P*, *A*	*H*, *M*
eNB insertion/removal	Self-configures home eNBs	*N*, *P*, *H*	-
CCO	Optimizes eNB coverage and capacity of eNB	*P*, *A*	*R*, *H*, *M*
Energy saving	Optimizes eNB energy consumption of eNB	*P*, *S*, *M*	-
MLB	Optimizes cell reselection/handover parameters	*P*, *A*, *S*, *H*	-
MRO	Automatically sets the handover parameters	*H*	*N*
Relay/repeater management	Self-configures and optimizes relays and repeaters	*R*, *P*, *S*	*H*, *M*

*N*—Neighbor list, *R*—Radio bearer assignment, *P*—Downlink Tx power, *A*–Antenna tilt and azimuth, *S*–Switching on/off cell, *H*—Handover parameters, *M*—MIMO configuration.

**Table 3:** The transition probabilities.

p	SON Function changes power to optimal value	$\sum_{i=1}^{N}\mathrm{Pr}\left(\phi_{i}\right)\times \mathrm{Pr}\left(P\;\right|{\;\phi }_{i})\times \mathrm{Pr}\left(O\right|\;\phi_{i}\cap P)$
a	SON Function changes antenna parameters to optimal value	$\sum_{i=1}^{N}\mathrm{Pr}\left(\phi_{i}\right)\times \mathrm{Pr}\left(A\;\right|{\;\phi }_{i})\times \mathrm{Pr}\left(O\right|\;\phi_{i}\cap A)$
h	SON Function changes handover parameters to optimal value	$\sum_{i=1}^{N}\mathrm{Pr}\left(\phi_{i}\right)\times \mathrm{Pr}\left(H\;\right|{\;\phi }_{i})\times \mathrm{Pr}\left(O\right|\;\phi_{i}\cap H)$
m	SON Function changes MIMO configuration to optimal value	$\sum_{i=1}^{N}\mathrm{Pr}\left(\phi_{i}\right)\times \mathrm{Pr}\left(M\;\right|{\;\phi }_{i})\times \mathrm{Pr}\left(O\right|\;\phi_{i}\cap M)$
p′	SON Function changes power to suboptimal value	$\sum_{i=1}^{N}\mathrm{Pr}\left(\phi_{i}\right)\times \mathrm{Pr}\left(P\;\right|{\;\phi }_{i}\times \mathrm{Pr}\left(S\right|\;\phi_{i}\cap P)$
a′	SON Function changes antenna parameters to suboptimal value	$\sum_{i=1}^{N}\mathrm{Pr}\left(\phi_{i}\right)\times \mathrm{Pr}\left(A\;\right|{\;\phi }_{i})\times \mathrm{Pr}\left(S\right|\;\phi_{i}\cap A)$
h′	SON Function changes handover parameters to suboptimal value	$\sum_{i=1}^{N}\mathrm{Pr}\left(\phi_{i}\right)\times \mathrm{Pr}\left(H\;\right|{\;\phi }_{i})\times \mathrm{Pr}\left(S\right|\;\phi_{i}\cap H)$
m′	SON Function changes MIMO configuration to suboptimal value	$\sum_{i=1}^{N}\mathrm{Pr}\left(\phi_{i}\right)\times \mathrm{Pr}\left(M\right|{\;\phi }_{i})\times \mathrm{Pr}\left(S\right|\;\phi_{i}\cap M)$

**Table 5:** Network setting with initial probabilities.

Probability of the network automation function to be selected	Equally Likely, 1/9
Probability of the primary parameter to be selected	70% of the time
Probability of any secondary parameter to be selected	30% of the time
Probability of a ANF to correctly configure any of its parameter (optimal value)	90% of the time
Probability of a ANF to misconfigure any of its parameter (suboptimal value)	10% of the time

**Table 6:** Description of the simulation parameters.

System Parameters	Value
Number of BSs	3
Number of Users	50
Operating frequency of BSs	1.7 GHz
Bandwidth of BSs	15 MHz
Pathloss exponent	3
Shadowing standard deviation	4 dB

## Data Availability

The original contributions presented in this study are included in the article. Further inquiries can be directed to the corresponding author.
